# Deciphering the Function of Octopaminergic Signaling on Wing Polyphenism of the Pea Aphid *Acyrthosiphon pisum*

**DOI:** 10.3389/fphys.2016.00603

**Published:** 2016-12-09

**Authors:** Xing-Xing Wang, Yi Zhang, Zhan-Feng Zhang, Hong-Gang Tian, Tong-Xian Liu

**Affiliations:** State Key Laboratory of Crop Stress Biology for Arid Areas, and Key Laboratory of Integrated Pest Management on the Loess Plateau of Ministry of Agriculture, Northwest A&F UniversityYangling, China

**Keywords:** neurotransmitter, RNA interference, tyramine β-hydroxylase, winged and wingless, alate, apterae

## Abstract

Aphids exhibit wing polyphenism (winged or wingless) for adaption to predictable or temporally heterogeneous environmental changes; however, the underlying mechanism is still unclear. This morphological change could be stimulated by high aphid density, which in turn could affect octopaminergic signaling in aphids. Octopamine is a neurotransmitter synthesized in insects that can modify their physiological metabolism, locomotion, and other behaviors. We designed experiments to determine whether octopamine functions in wing formation of the pea aphid, *Acyrthosiphon pisum* (Harris). We determined gene expression of tyramine β-hydroxylase (*T*β*H*), a key enzyme in octopamine synthesis at different developmental stages, in different body parts, and in different densities of aphids. We also used *T*β*H* RNAi, octopamine receptor agonists (octopamine and synephrine), and an antagonist (mianserin) to modify octopaminergic signaling. We found that transcription of *T*β*H* was related to aphid density, which affected the proportion of winged offspring. By manually modifying the mother's octopaminergic signaling, *T*β*H* expression was suppressed, and TβH (enzyme) activity decreased. The proportion of winged offspring was also affected. Our results showed that octopamine could be a link in the wing determination system, as well as environmental stimulation. The RNAi results showed that the decrease of *T*β*H* expression increased aphid's reproduction; however, the decrease of *T*β*H* expression declined the numbers of winged-offspring producers, but did not affect the proportion of winged nymphs produced by the winged-offspring producer. In conclusion, the decline in the proportion of winged daughters in the next generation was caused by the decline of winged nymph producers.

## Introduction

The pea aphid, *Acyrthosiphon pisum* (Harris), shows polyphenism in winged (alatae) and wingless (apterae) morphs (Braendle et al., 2006). For parthenogenetic females, density, in particular, its effects on tactile stimulation and nutrition (host plant quality), is considered to be the key factor influencing the production of winged or wingless daughters (Braendle et al., 2006; Brisson, 2010). The winged and wingless aphids exhibit differences in morphological, physiological, and behavioral features. Winged individuals have functional flight muscles and other characteristics relating to flight for dispersal, whereas wingless individuals are primarily sedentary and walk only short distances. Normally, the wingless morph has a relatively larger abdomen and greater nymphal production capacity than the winged morph (Sack and Stern, 2007).

Wing formation typically occurs under unfavorable abiotic and biotic conditions. For example, increasing aphid density triggers wing formation in their offspring in many aphid species, which could be caused by an increased tactile stimulation among individuals under high density (Lees, 1967; Sutherland, 1969). Some studies have suggested that the stimulation receptors are located on the antennae, although the underlying physiological mechanism is still unknown (Lees, 1967; Sutherland, 1969; Braendle et al., 2006). Host plant quality or nutrition is also considered an important stimulus for winged aphid production; however, the connection between nutrition and winged offspring formation is still unclear. It is possible that poor nutrition could increase physical contact between aphids (Müller et al., 2001). It has been shown that their natural enemies, ants (mutualism), and aphid endosymbionts could also affect production of winged offspring (Johnson, 1966; Sutherland and Mittler, 1971; Wratten, 1977).

Maternal factors could affect wing determination of offspring. In many aphid species, especially the pea aphid, wing determination occurs prenatally; hence, aphids themselves cannot influence their wing morph. Conversely, winged mothers rarely produce winged daughters, even under wing-stimulating conditions (MacKay and Wellington, 1976; Brisson, 2010). These findings suggest that pea aphids cannot change their phenotype within one generation in response to environmental changes (Brisson, 2010).

It has been shown that high density (tactile stimulation) strongly affects wing formation frequency in pea aphids, but the details of the physiological mechanism are still unclear (Braendle et al., 2006; Brisson, 2010). Studies on juvenile hormone (JH) in aphids produced conflicting results, and to date no study has successfully correlated JH titers with the production of wingless morphs (Hardie, 1980, 1981; Hardie et al., 1995, 1996). Wing formation signals in aphids and the mechanism of the wing formation process requires further study (Applebaum et al., 1975; Hardie et al., 1995; Gao and Hardie, 1996).

Octopamine is a neurohormone, neuromodulator, and neurotransmitter in many invertebrates including insects (Scheiner et al., 2002). It acts as an insect equivalent of norepinephrine and mobilizes the body and nervous system of insects (Roeder, 1999). Many behaviors, such as rhythms, feeding, flying, courtship, and aggression are all modified by octopamine in insects (Long and Murdock, 1983; O'Dell, 1993; Orchard, 1993; Cohen et al., 2002; Pophof, 2002; Mentel et al., 2003; Monastirioti, 2003; Zhou et al., 2008). Learning and memory in insects also require octopamine (Hammer and Menzel, 1998; Schwaerzel et al., 2003). It has been shown that octopamine is related to alcohol tolerance in fruit flies, light production in fireflies, and phase change regulation in locusts (Christensen et al., 1983; Scholz et al., 2000; Ma et al., 2015). Octopamine is synthesized in insects from tyramine by β-hydroxylation (tyramine β-hydroxylation, TβH). Tyramine, the precursor of octopamine, is synthesized from tyrosine by tyrosine decarboxylase (TDC), and tyramine itself is a neurotransmitter in invertebrates (Walker and Kerkut, 1978; Roeder, 1999). As a key enzyme in octopamine biosynthesis, *T*β*H* modification and detection can be used for octopamine regulation and level analysis (Certel et al., 2007; Nishimura et al., 2008; Chen et al., 2012; Wu et al., 2013).

Physical contacts (tactile stimulation) caused by crowding (high density) or poor nutrition could increase dispersal of aphids. We assumed that octopamine might function in the wing-formation control system, and could affect wing formation of embryos inside the female adults. In this study, we first analyzed wing formation frequency at high densities, and then analyzed *T*β*H* expression patterns at different aphid densities. We also used RNAi and octopamine receptor agonists and an antagonist to modify *T*β*H* expression to determine the function of octopamine in wing formation of aphid offspring.

## Materials and methods

### Aphids and plants

A red strain of pea aphid was collected from Gansu Province, China, and cultured on broad bean (*Vicia faba* L., var. “Jinnong”) under a long-day photoperiod (16L: 8D; 20 ± 1°C) for more than 30 generations at the Key Laboratory of Applied Entomology, Northwest A&F University, China. All wingless aphids were reared at a low density (less than 30 aphids per 4-week-old seedling) for more than three generations before they were used in subsequent experiments. A high density (30 individuals per 2-week-old plant seedling) was used to stimulate wing formation. All winged aphids were selected and reared at a low density (less than 30 individuals per 4-week-old seedling) before they were used in all experiments. All adult aphids used for analysis were 36-h old.

### Transcription analysis of *TβH*

To analyze expression differences in the rate-limiting enzyme *T*β*H* of octopamine, aphid samples were frozen immediately after collection using liquid nitrogen. RNA was extracted with RNAiso Plus (Takara, Tokyo, Japan), and cDNA was synthesized using a PrimeScript™ RT reagent kit with gDNA Eraser (Takara, Tokyo, Japan). Quantitative real-time PCR (qRT-PCR) was performed with SYBR® Premix Ex Taq™ II (Takara, Tokyo, Japan) in an IQ-5 system (Bio-Rad, Berkeley, CA, USA). The primers were designed by Primer-BLAST of NCBI online (http://www.ncbi.nlm.nih.gov/tools/primer-blast/index.cgi?LINK_LOC=BlastHome) and the positions of sequences are shown in **Figure 2A**. *Rpl7* (F: GCGCGCCGAGGCTTAT, R: CCGGATTTCTTTGCATTTCTTG) was selected as a reference gene for our Q-PCR experiments (Nakabachi et al., 2005).

### Expression modification of *TβH*

The dsRNA of *T*β*H* in the octopamine metabolic pathway was prepared using the T7 RiboMAX system (Promega). Then, 200 nL of dsRNA (6000 ng μL^−1^) from target genes or a non-aphid related gene *lta-mus* was injected as a control. The primers used for synthesis of two designed dsRNAs are shown in **Figure 2A**. The control dsRNA was selected from *lymphotoxin A* of *Mus musculus* (*lta-mus*, Gene ID: 16992). The primers used for *lta-mus* dsRNA synthesis were F-CACCCTCTCCACGAATTG and R-TAGAAGATGCTGCTGTTTCA.

*T*β*H* dsRNA was injected as described by Barron et al. (2007), Scheiner et al. (2002); Zhang et al. (2016) and Chen et al. (2016) with modification. A glass needle, made from a glass capillary (3.5 inc 3-000-203-G/X micropipettes, Drummond Scientific, Broomall, PA, USA) by a micropipette puller (P-97 Micropipette Puller, Sutter, CA, USA; pulling program: Pull = 100, VEL = 100, and Time = 100), was used with the Nanoject II Auto-Nanoliter Injector (Drummond Scientific Company, Broomall, PA, USA) for injection. The *T*β*H* dsRNA solution (ds-*T*β*H1*, ds-*T*β*H2*, and ds-*T*β*H1*+ *T*β*H2*; 6000 ng μL^−1^) or octopamine (1 mg mL^−1^), synephrine (0.1 mg mL^−1^), and mianserin (0.1 mg mL^−1^) solution were separately injected into the ventral thorax through the fissure at the base of the hind leg (T3 segment) of the aphids (**Figure 2B**); 200 nL solution per aphid was injected. The aphids were then placed on a *V. faba* seedling, and were reared under a low density for further winged-offspring stimulation or transcription analysis.

### Experimental design

#### *TβH* expression in the head and abdomen of different stages and morphs

**Figure 1:**
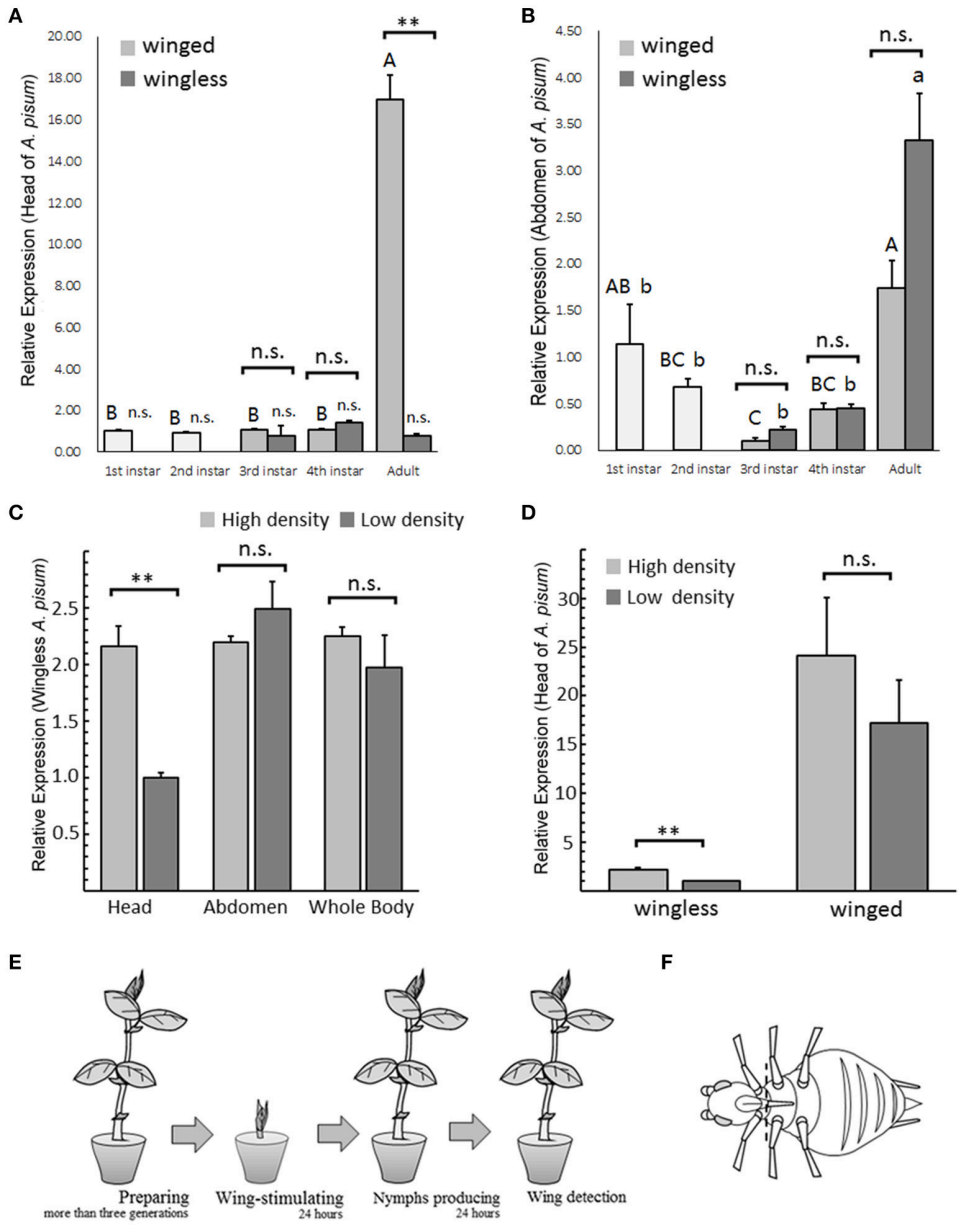
*****T***β***H*** expression patterns in ***Acyrthosiphon pisum*** under different conditions**. *T*β*H* expression analysis in different stages of winged and wingless *A. pisum*, were detected separately in the head **(A)** and in the abdomen **(B)**. The analysis of *T*β*H* expression in head, abdomen and whole body of wingless *A. pisum* raised at different densities **(C)** and *T*β*H* expression difference in heads between winged and wingless morphs **(D)**. ^**^ in **(A–D)** indicate significantly different at *P* < 0.01, respectively (Student's *t*-test), and different letters in **(A,B)** on top of the bars indicate significantly differences (*P* < 0.05, Duncan's test). The schematic of winged-stimulation protocol based on densities **(E)** and dissection positions (dotted line) in *A. pisum* are shown in **(F)**.

Winged and wingless aphids at each stage under low-density conditions (less than 30 individuals per 4-week-old seedling) were prepared. These aphids were dissected between the T1 and T2 segments (Figure 1F) in liquid nitrogen. All prepared samples (head with the T1 segment; abdomen with the T2 and T3 segments) were kept at −80°C until used for *T*β*H* expression analysis. Three independent replicates of 50 first or second instar nymphs and 15 third or fourth instar nymphs or adults were prepared.

#### *TβH* expression in different aphid densities

Winged and wingless adult aphids under low-density conditions (less than 30 individuals per 4-week-old seedling) were prepared for experiments. All selected aphids were kept on newly germinated *V. faba* plants (7–10 days old, 1.5 cm in length). For winged-nymph stimulation, 30 individuals were placed on each plant as a crowding stimulus, and the stimulation lasted 24 h (Figure 1E).

After wing-formation stimulation, the wingless aphids were dissected between the T1 and T2 segments (Figure 1F) in liquid nitrogen. The head with T1 segment and abdomen with T2 and T3 segments were used for *T*β*H* expression analysis. Un-dissected aphids were also prepared for analysis. Three independent replicates of 15 aphids were prepared.

To analyze *T*β*H* expression differences between the winged and wingless aphids, the two aphid morphs were separately dissected between the T1 and T2 segments (Figure 1F) in liquid nitrogen after wing formation stimulation. The head (with T1 segment) was used for *T*β*H* expression analysis. Three independent replicates of 15 aphids each were used.

#### RNA interference of *TβH* in *A. pisum*

##### RNA interference in head and abdomen

Eighty wingless adult aphids under low-density conditions (less than 30 individuals per 4-week-old seedling) were prepared for dsRNA injection (ds-*T*β*H1*, ds-*T*β*H2*, and ds-*lta-mus*) as described above, and non-treated aphids were prepared to serve as controls. The injected aphids were replaced on a *V. faba* seedling at low density and reared for 24 h before used for high-density stimulation. One-half of the aphids were collected and dissected between the T1 and T2 segments and both parts were used for *T*β*H* expression analysis. The remaining aphids were used whole for *T*β*H* expression analysis. Three independent replicates of 15 aphids were prepared.

##### Repression abilities of our designed TβH dsRNA

Wingless adult aphids under low-density conditions (less than 30 individuals per 4-week-old seedling) were prepared for dsRNA injection (ds-*T*β*H1*, ds-*T*β*H2* ds-*T*β*H1*+ *T*β*H2*, and ds-*lta-mus*) as described above and non-treated aphids were prepared to serve as controls. The injected aphids were replaced on a *V. faba* seedling at low density (less than 30 individuals per 4-week-old seedling) and reared for 6 days. Thereafter, aphid samples were collected daily. The aphids were dissected between the T1 and T2 segments for *T*β*H* expression analysis. Three independent replicates of 15 aphids were prepared.

##### TβH expression in offspring of dsRNA treated *A. pisum*

Sixty wingless adult aphids under low-density conditions (less than 30 individuals per 4-week-old seedling) were prepared for dsRNA injection (ds-*T*β*H1*, ds-*T*β*H2*, and ds-*lta-mus*) as described above and non-treated aphids were prepared and served as controls. The injected aphids were replaced on *V. faba* seedlings at low density (less than 30 individuals per 4-week-old seedling) and reared for 3 days. The offspring were collected and immediately placed in liquid nitrogen and kept frozen (−80°C) until use. Samples were collected daily (day 1: 12–18 h after injection, day 2: 36–42 h after injection, and day 3: 60–66 h after injection; only newborn aphids were collected) and used for *T*β*H* expression analysis. Three independent replicates of 30 nymphs were prepared.

#### TβH assay

##### TβH assay protocol

**Figure 2:**
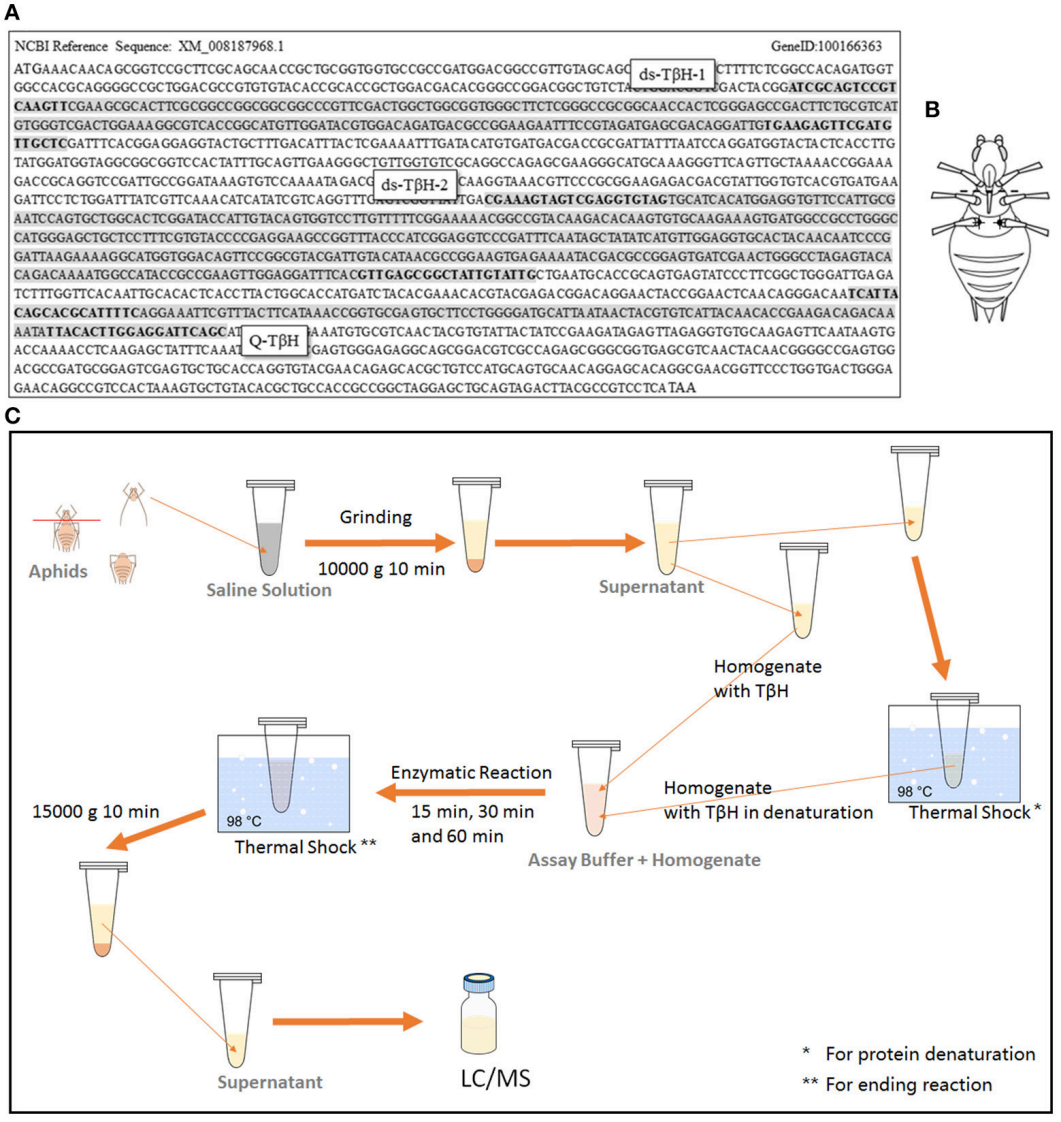
**Sequence of ***T***β***H*** in ***Acyrthosiphon pisum***, and two designed dsRNA were marked (positions of primers were marked in bold); fragment for qRT-PCR test was also marked in the sequence (A)**. Dissection (dotted line) and injection (dark spots) position are shown in **(B)**, and the TβH assay protocol is shown in **(C)**.

The TβH assay was based on the hydroxylation of tyramine (Lehman et al., 2000a,b; Châtel et al., 2013; Figure 2C). All steps were performed in the dark or under dim light. Aphids were dissected between the T1 and T2 segments (Figure 1F) in liquid nitrogen, and the head (with T1 segment) was used for TβH assay. The samples were immediately placed in ice-cold saline solution (150 mM NaCl, 3 mM KCl, 3 mM CaCl_2_, 20 mM MgCl_2_, and 10 mM N-Tris [hydroxymethyl] methyl-2-aminoethanesulfonic acid, pH 6.9) (Christensen et al., 1992). The tissues were frozen in liquid nitrogen and stored at −80°C. After weighing, the tissues were homogenized in saline solution (500 μL per 5 mg tissue) using a small plastic pestle in a centrifuge tube. The homogenate was centrifuged (10 min, 10,000 g, 4°C). The supernatant containing the enzyme extract was collected and kept frozen (−80°C) until use. The homogenates were equally split, and one of them was prepared by thermal shock (5 min, 98°C) for denaturation of protein as a control. All samples were added (final concentration 250 mg mL^−1^) in assay buffer (0.1 M potassium phosphate, pH 6.9, 1 mg mL^−1^ catalase, 0.1 mM N-ethylmaleimide, 0.05 mM CuSO_4_, 5 mM disodium fumarate, 5 mM ascorbic acid, and 0.05 mM tyramine) and incubated at room temperature for 15, 30, and 60 min without shaking. The reaction was immediately stopped by thermal shock (5 min, 98°C). The samples were centrifuged at 15,000 g for 10 min, and the supernatant was collected and used for LC/MS analysis. The TβH assays were performed in six independent experiments.

##### Tyramine and octopamine assays

**Figure 3:**
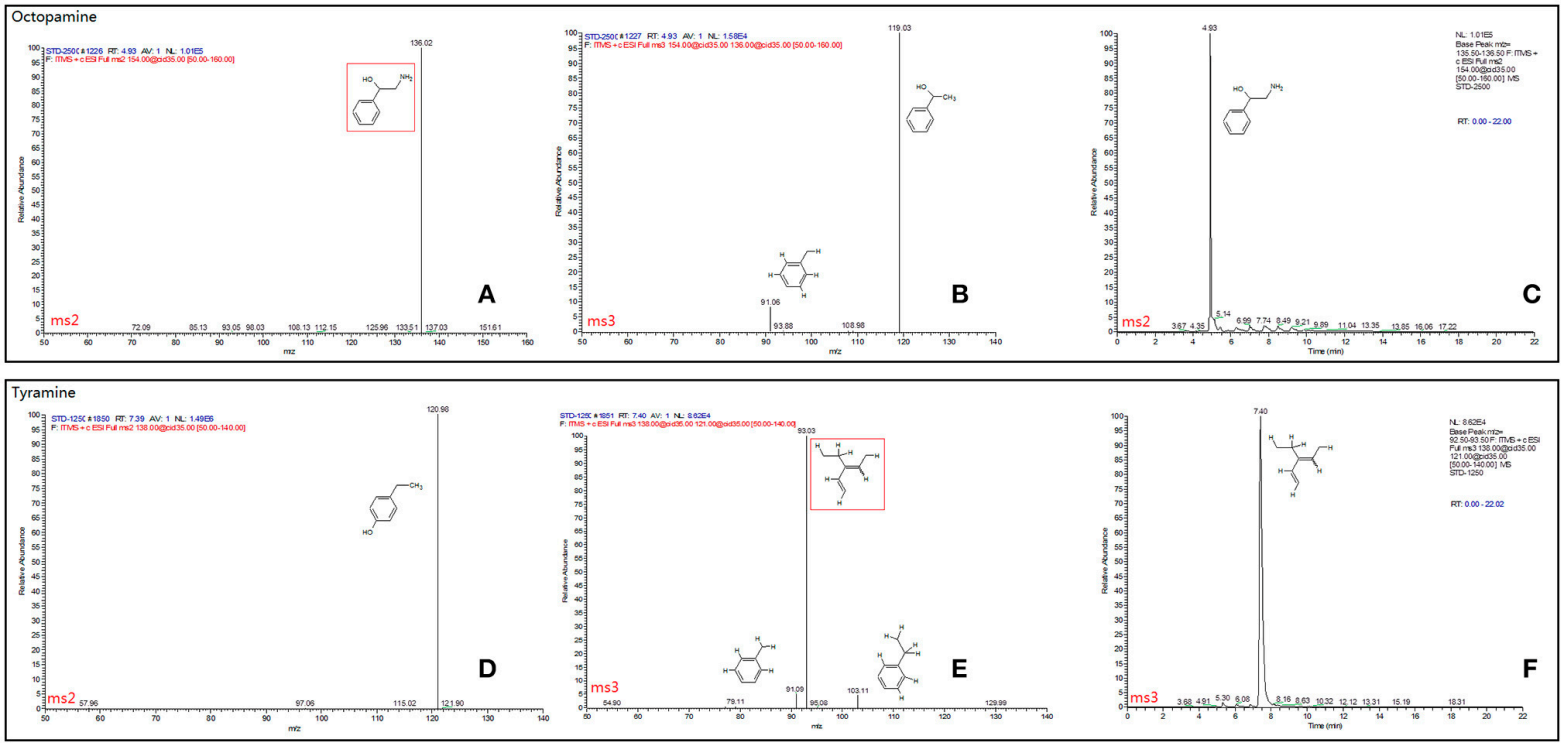
**Full daughter scan MS Spectra and selected ion retention time (min) of octopamine (A–C)** and tyramine **(D–F)**. The mass spectrometer was set in the positive electrospray ionization mode. Nitrogen was used as the sheath gas (40 arbitrary units) and auxiliary gas (10 arbitrary units). The spray voltage was set at 4.5 kV and the ion transfer capillary temperature was 275°C. MS Spectra information was referred to the database of METLIN (Scripps Center for Metabolomics METLIN: Metabolite and Tandem MS Database http://metlin.scripps.edu/terms.php).

The final samples described above for TβH assays were analyzed by an LTQ XL linear ion trap mass spectrometer (Thermo-Fisher Scientific, Waltham, MA, USA). Tyramine and octopamine were scanned and fragmented using data dependent MS/MS. Masses of precursor and productions for each chemical and full daughter scan MS Spectra and selected ion retention time (min) are shown in Figure 3. All data were acquired and processed using Xcalibur 2.1 software (Thermo-Fisher Scientific, Waltham, MA, USA). Quantification was achieved using external standard tyramine and octopamine mixtures of known concentration.

#### Nymph production and winged offspring formation in *A. pisum* under octopaminergic signaling modifications

##### Nymph production analysis

Wingless adult aphids at low density were prepared for dsRNA injection (ds-*T*β*H1* and ds-*lta-mus*) as described above. For further analysis, octopamine, synephrine (octopamine receptor agonist), and mianserin (octopamine receptor antagonist) were prepared for aphids injection (1 mg mL^−1^), and un-injected and saline-injected aphids were prepared as controls. Fifty injected aphids were replaced on leaves of *V. faba* seedlings and individually reared for 7 days (leaves were kept in 1% agar in a 24-well plate and replaced by new leaves every 2 days). The nymphs' production of each aphid was determined daily and the offspring were removed.

##### Winged offspring formation

Wingless adult aphids at low density (less than 30 individuals per 4-week-old seedling) were prepared for injection (ds-*T*β*H1, lta-mus*, octopamine, synephrine, and mianserin and saline) and un-injected aphids were prepared as controls. The dsRNA was injected as described above, and 50 aphids were used. All injected aphids were replaced on *V. faba* seedlings and reared for 24 h at low density (less than 30 individuals per 4-week-old seedling). The treated aphids were transferred to newly germinated *V. faba* (7–10-days old, 1.5 cm in length) as described above. The aphids were then reared separately in a 24-well plate as described above after wing stimulation for 24 h. Mothers were removed after 24 h of production and the nymphs produced during 24 h were kept and reared separately in a 24-well plate. The preparations of winged nymphs were counted after they developed into the third instar when winged or wingless individuals could be distinguished.

### Statistical analysis

The *T*β*H* expression data in the head and the abdomen between the winged and wingless aphid morphs among different developmental stages, and the data of *T*β*H* expression in the head and the abdomen of adults between the high and low densities were subjected to Student's *t*-test at *P* < 0.05. The *T*β*H* expression data among different developmental stages of each morph were subjected to one-way analysis of variance (ANOVA); means were separated using Duncan's test at *P* < 0.05.

The *T*β*H* expression data under RNAi in the head and the abdomen of wingless adults, mothers' reproduction data under RNAi, and the data of *T*β*H* bioassays were subjected to ANOVA; means were separated using Duncan's test at *P* < 0.05. The proportion data of winged offspring under different treatments were analyzed using one-way ANOVA, and the proportions of winged producers under treatments were separated by Chi-square test (*P* < 0.05). SPSS Version 22; SPSS Inc., Chicago, IL, USA) was used for data analysis.

## Results

### *TβH* expression in different aphid stages

*T*β*H* transcription level differed only in the head of the adults, and *T*β*H* showed up-regulation in winged adults (*t* = 12.653, *df* = 4, *P* = 0.005; Figure 1A). All the other tests failed to detect any difference (head: third instar, *t* = 0.537, *df* = 4, *P* = 0.620; fourth instar: *t* = −2.853, *df* = 4, *P* = 0.086, Figure 1A; abdomen: third instar, *t* = −2.347, *df* = 4, *P* = 0.076; fourth instar: *t* = −0.084, *df* = 4, *P* = 0.937; adult: *t* = −2.685, *df* = 4, *P* = 0.055; Figure 1B).

*T*β*H* expression at different stages showed significant differences. Gene expression in adult's head was extremely high in the winged aphids (*F* = 184.189, *df* = 4, 10, *P* < 0.0001; Figure 1A), whereas the expression in the wingless aphids was relatively stable during development (*F* = 1.295, *df* = 4, 10, *P* = 0.336; Figure 1A). *T*β*H* was highly expressed in the adult abdomen in both aphid morphs (winged, *F* = 7.313, *df* = 4, 10, *P* = 0.005; wingless, *F* = 17.23, *df* = 4, 10, *P* < 0.0001; Figure 1B).

### *TβH* expression differences in aphids living at different densities

Regarding *T*β*H* expression in the head of wingless aphids, the aphids under high-density conditions exhibited significantly higher up-regulation than those under low-density conditions (*t* = 6.476, *df* = 4, *P* = 0.003; Figure 1C). No expression differences between the two densities were found in the abdomen (*t* = −1.154, *df* = 4, *P* = 0.313; Figure 1C) or the whole body (*t* = 0.923, *df* = 4, *P* = 0.408; Figure 1C).

*T*β*H* in the heads of the wingless aphids reared under high-density conditions was more significantly up-regulated compared to wingless aphids reared under low-density conditions (*t* = 6.476, *df* = 4, *P* = 0.003; Figure 1D), and no changes were observed in the winged aphids (*t* = 0.935, *df* = 4, *P* = 0.403; Figure 1D).

### *TβH* expression modification by RNAi

**Figure 4:**
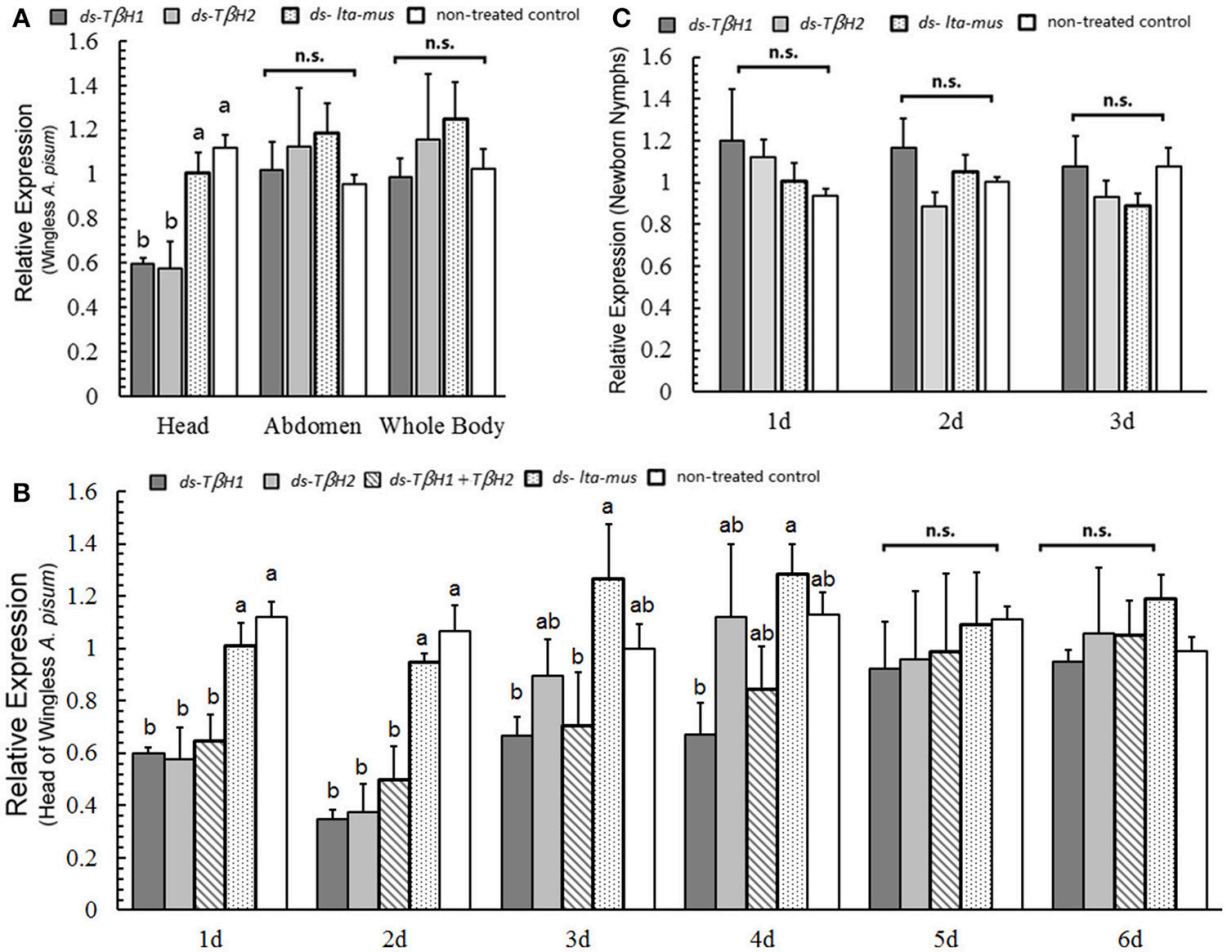
*****T***β***H*** expression under RNAi experiments in wingless ***Acyrthosiphon pisum***.** *T*β*H* expression in the head, abdomen and whole body of wingless *A. pisum* under *T*β*H* RNAi **(A)**; *T*β*H* expression (two dsRNA) in the head after dsRNA repression for 6 days **(B)**, and *T*β*H* expression in the newborn nymphs after *T*β*H* RNAi of their mothers for 3 days **(C)**. Each value represents the mean ± SEM, and different letters in a and b on top of the bars indicate significantly different (*P* < 0.05, Duncan's test).

Following the injection of the two designed *T*β*H* dsRNAs into *A. pisum*, reduction in *T*β*H* expression was not observed in the abdomen (*F* = 0.411, *df* = 3, 8, *P* = 0.750; Figure 4A) or whole body (*F* = 0.462, *df* = 3, 8, *P* = 0.717; Figure 4A). However, *T*β*H* was suppressed by the two *T*β*H* dsRNAs in the heads (*F* = 11.544, *df* = 3, 8, *P* = 0.003; Figure 4A).

The dsRNAs reduced the expression of *T*β*H* in the first three days (day 1, *F* = 8.793, *df* = 4, 10, *P* = 0.003; day 2, *F* = 14.217, *df* = 4, 10, *P* < 0.0001; day 3, *F* = 2.469, *df* = 4, 10, *P* = 0.112; Figure 4B). Later, expression of *T*β*H* was indistinguishable from that of control individuals in the following 3 days (day 4, *F* = 2.219, *df* = 4, 10, *P* = 0.140 day 5, *F* = 0.146, *df* = 4, 10, *P* = 0.961; day 6, *F* = 0.446, *df* = 4, 10, *P* = 0.773; Figure 4B). Based on these results, *T*β*H1* dsRNA was slightly more effective than *T*β*H2* dsRNA and the combination of both (*T*β*H1*+ *T*β*H2*) dsRNAs.

The offspring of *T*β*H*-dsRNA injected *A. pisum* did not exhibit any up- or down-regulation in *T*β*H* expression three days after injection (day 1, *F* = 0.738, *df* = 3, 8, *P* = 0.558; day 2, *F* = 1.739, *df* = 3, 8, *P* = 0.236; day 3, *F* = 0.932, *df* = 3, 8, *P* = 0.469; Figure 4C). *T*β*H* expression in the offspring of all treated aphids was similar.

### Mother reproduction abilities following injection treatments

**Figure 5:**
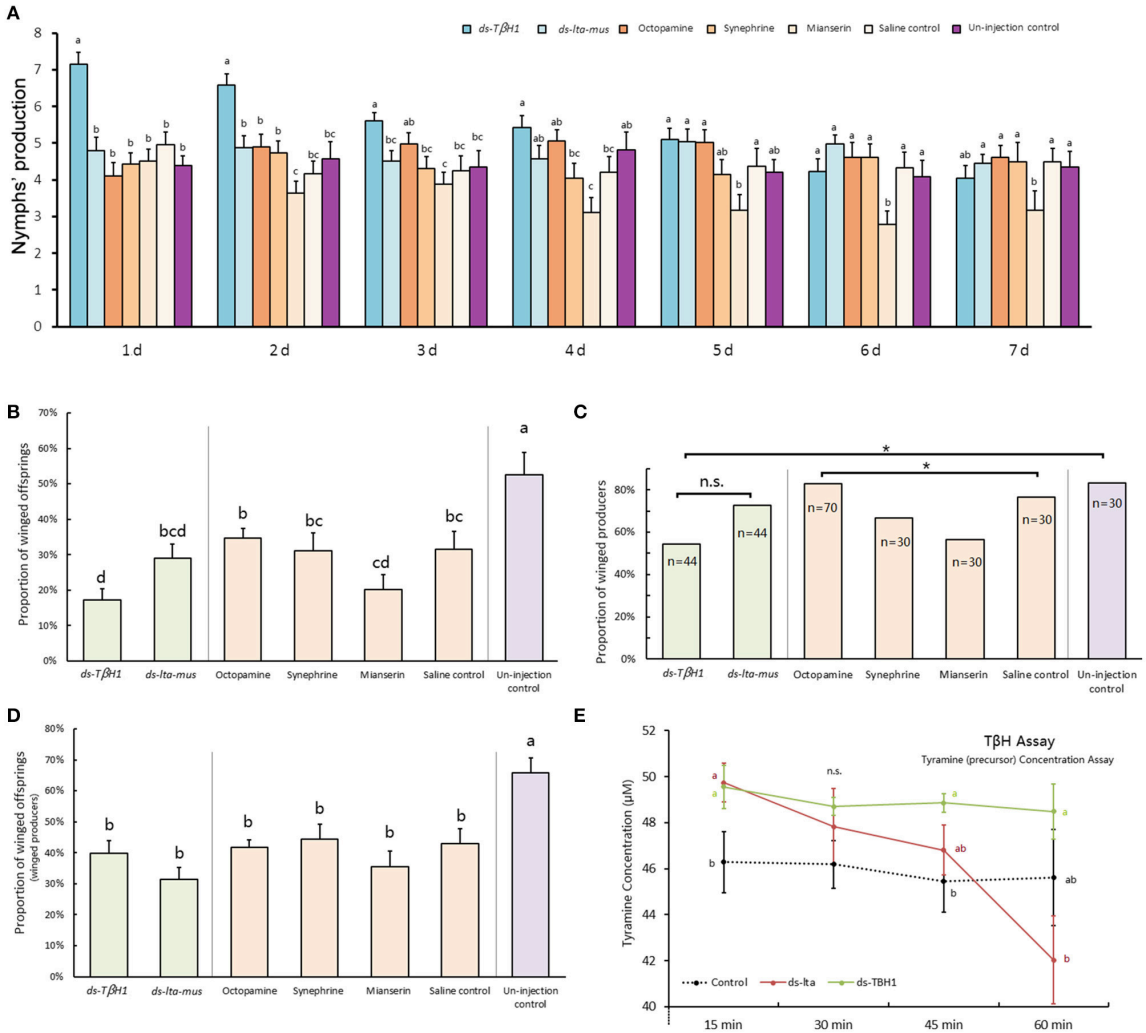
**dsRNA, octopamine, octopamine receptor agonist and antagonist injection in ***Acyrthosiphon pisum***.** Reproduction abilities changes of *A. pisum* 7 days after injection **(A)**; proportions of winged offspring of each aphid after injection **(B)**; proportions of winged producers of all experimental aphids after injection **(C)**; proportions of winged offspring produced by winged producers after injection **(D)**; and tyramine concentration difference in TβH bioassays under RNAi **(E)**. Each value represents the mean ± SEM, different letters in **(A,B,D,E)** at the bars or points indicate significantly different (*P* < 0.05, Duncan's test, and the letters in **(E)** show comparison of three treatments at each reaction time) and ^*^ of **(C)** indicates significant different based (*P* < 0.05, χ^2^-test).

Reproduction abilities of *A. pisum* increased after *T*β*H* dsRNA injection and decreased after mianserin injection. The *T*β*H1* dsRNA treated aphids produced significantly more offspring than did those in other treatments in the first 3 days and beginning on the second day following the injection, and mianserin led to a significantly reduced number of nymphs produced (day 1: *F* = 10.662, *df* = 6, 233, *P* < 0.0001; day 2: *F* = 7.808, *df* = 6, 232, *P* < 0.0001; day 3: *F* = 3.612, *df* = 6, 232, *P* = 0.002; day 4: *F* = 3.963, *df* = 6, 232, *P* = 0.001; day 5: *F* = 3.351, *df* = 6, 214, *P* = 0.004; day 6: *F* = 2.934, *df* = 6, 205, *P* = 0.009; day 7: *F* = 1.517, *df* = 6, 195, *P* = 0.174; Figure 5A).

The reproduction abilities of *A. pisum* injected with *T*β*H1* dsRNA decreased (*F* = 8.532, *df* = 6, 266, *P* < 0.0001) days after injection, whereas that of the ds-lta control aphids was relatively consistent (*F* = 1.997, *df* = 6, 229, *P* = 0.067). The reproduction of *A. pisum* injected with mianserin significantly decreased (*F* = 2.467, *df* = 6, 151, *P* = 0.026).

### TβH assay

Octopamine was not detected, and its precursor (tyramine) was used for TβH bio-assays (Supplementary Data Sheet). In comparison with the non-enzyme treatment (protein denatured by thermal shock) for the 15-min treatment, tyramine concentrations in the two treatments were significantly higher than those of non-enzyme control. The tyramine levels were similar between the two samples treated by *lta* and *T*β*H1* dsRNAs (*F* = 3.347, *df* = 2, 15, *P* = 0.063; Figure 5E).

The levels of tyramine declined during the enzymatic reaction in both the ds-lta and ds-TβH1 treatments, whereas tyramine concentration was relatively stable in the control. Regarding treatments with ds-TβH1, tyramine levels of ds-lta treated samples declined more sharply than those of ds-TβH1 treated samples (30 min: *F* = 1.238, *df* = 2, 15, *P* = 0.318; 45 min: *F* = 2.884, *df* = 2, 15, *P* = 0.087; Figure 5E), and the concentrations were significantly different for the 60-min treatment (*F* = 3.354, *df* = 2, 15, *P* = 0.063; Figure 5E).

### Winged offspring production analysis

The proportion of winged offspring of *A. pisum* declined after the mothers were treated with *T*β*H* dsRNA and mianserin for 24 h under high-density conditions. *A. pisum* treated with *T*β*H* dsRNA produced significantly fewer winged offspring (17.19%) than those in the ds-lta control (29.0%). Octopamine, synephrine, and mianserin injections revealed that treatment with mianserin (octopamine receptor antagonist) resulted in fewer winged nymphs (20.1%). Compared with the saline control (31.5%), winged nymph productions following octopamine (34.6%) and synephrine (31.2%, octopamine receptor agonist) treatments were not significantly altered. The mothers that did not receive injections (non-treated control) had the highest numbers of winged offspring among all treatments (52.6%) (*F* = 6.769, *df* = 6, 271, *P* < 0.0001; Figure 5B).

Comparing with the *lta* dsRNA treatment (72.7%), relatively fewer mothers in the *T*β*H* dsRNA (54.6%) treatments were successfully stimulated and turned into winged-offspring producers (χ^2^ = 3.143, *df* = 1, *P* = 0.076; Figure 5C). Similarly, fewer mianserin (56.67%) treatments were successfully stimulated than the mothers treated with octopamine (82.9%), synephrine (66.67%), and the saline control (76.67%) (χ^2^ = 8.431, *df* = 3, *P* = 0.038; Figure 5C). For all aphids, the highest proportion of winged-offspring producers was observed in untreated control (83.33%) (χ^2^ = 16.736, *df* = 6, *P* = 0.010; Figure 5C).

The proportions of winged offspring by winged-offspring producer in all treatments were similar except for the non-treated control which produced a significantly higher proportion of winged nymphs (*F* = 6.390, *df* = 6, 191, *P* < 0.0001; Figure 5D). No winged offspring was observed in the low-density treatments.

## Discussion

Our results confirmed that octopaminergic signaling functions in wing polyphenism of parthenogenetic females of the pea aphid. Expression of the key enzyme octopamine *T*β*H* differed in different morphs and different developmental stages, and *T*β*H* expression varied in the wingless *A. pisum* adults that were exposed to different densities. It was also shown that *T*β*H* expression could be suppressed by *T*β*H* dsRNA and the suppression lasted for more than 3 days. Combined with the results of the experiments on the octopamine receptor antagonist and agonist, nymph production and winged offspring were significantly affected by octopaminergic signaling modification.

Compared with winged parthenogenetic *A. pisum* females, *T*β*H* expression in the head of wingless adults was lower. Because winged aphids were normally more active than the wingless aphids, locomotion differences might be the link that connects *T*β*H* expression and wing formation. The difference in expression of *T*β*H* was only observed in the head of the aphid, whereas no differences were found in the abdomen, which is filled with embryos. *T*β*H* expression of embryos would influence transcription level detection in mother aphids. Density experiments indicated that *T*β*H* expression in heads was affected by tactile stimulation, which is similar to that of the migratory locust (*Locusta migratoria* L.) (Ma et al., 2015). A high density of aphids could make aphids more active, resulting in more body contact. Based on this, we assumed that octopaminergic signaling connected wing formation signal and physical contact. Transcription analysis of *T*β*H* from the whole aphid body did not reveal any difference for aphids at different densities. We believe that the high proportion of embryos balanced the expression levels between the treatments and the control. That *T*β*H* expression was up-regulated in the head in high-density treatments provided the strongest evidence. Considering these results, octopaminergic signaling in aphid embryos seems unaffected by the tactile stimulation experienced by their mothers. Compared with wingless adults, *T*β*H* expression in winged aphid heads was always at a relatively high level (Figure 1D). Aphid's high density apparently affected octopaminergic signaling (*T*β*H* expression), but the difference was not significant. We suggest that the winged and wingless adults could have different wing-determination systems downstream.

In our RNAi experiments, we found that *T*β*H* expression could be suppressed, and the suppression lasted at least for 3 days. This result was similar to the results of previous RNAi studies on aphids (Mutti et al., 2006; Jaubert-Possamai et al., 2007; Zhang et al., 2013; Christiaens et al., 2014). The two designed *T*β*H* dsRNAs differed in suppression abilities, and the *T*β*H1* dsRNA-induced suppression lasted longer than the *T*β*H2* effect and even longer than that of the mixture of *T*β*H1*+*2* dsRNA. It seems that the suppression abilities are related to sequence location. Some segments of mRNA could be easily silenced by dsRNA. More easy-silenced segments that one dsRNA contained provided stronger RNA interference in the aphid.

Compared with *T*β*H* down-regulation in the head of *A. pisum*, there was no obvious reduction in the abdomen. Although previous studies reported that the RNAi response could be found in the embryos or offspring of aphids (Mutti et al., 2006; Shakesby et al., 2009; Pitino et al., 2011; Zhang et al., 2013; Shang et al., 2016), our results indicated that the dsRNA may not be able to enter embryos because no *T*β*H* repression was detected in the abdomen. Similarly, no significant transcript reduction of *T*β*H* was found in the newborn nymphs when their mothers were injected with the corresponding dsRNA (Figure 4C). Considering the high production of embryos by an aphid, this could explain why we did not detect gene repression in previous RNAi experiments of whole body and abdomen tests. In *L. migratoria*, the ovary appears to be less sensitive to RNAi than other tissues (Ren et al., 2014). It is possible that there was a similar barrier in embryos of the pea aphid. This result also helps us understand wing formation of the pea aphid, where octopaminergic signaling might be important only in the mother's generation, and wing formation of the nymphs could be affected.

In the present study, the direct assay of octopamine in the aphid body was not successful, and no signal was detected. The newly generated octopamine in the TβH assay samples was also not detected, and therefore, analysis of the precursor (tyramine) was used to reflect TβH activity. The results revealed that tyramine concentrations in the samples with functional proteins were higher than in the control (protein denatured by thermal shock). It is possible that the mixture contained functional tyrosine decarboxylase and that this enzyme was still active and new tyramine was generated. Of the two dsRNAs in the treated aphids, the ds-TβH1 treated samples had weaker tyramine β-hydroxylation than the samples treated with ds-lta (Figure 5E). This result suggested that *T*β*H* RNAi successfully affected synthetase activity in *A. pisum*.

Octopamine is involved in oviposition in *Drosophila melanogaster* (Monastirioti et al., 1996; Gruntenko et al., 2007). We found that parthenogenic nymph production of *A. pisum* might also be related to octopaminergic signaling, and that *T*β*H* down-regulation lead to more *A. pisum* offspring as compared with the control. Because a high level of octopamine could increase the locomotion of *A. pisum*, it is possible that down-regulated *T*β*H* lowers the level of octopamine in aphids and they became more sedentary and produced more nymphs than those in the control. On the other hand, our data showed that octopamine and synephrine (octopamine receptor agonist) injections did not reduce offspring. Compared with the ds-TβH1 treatment, the nymphs' production decreased in aphids injected with an octopamine receptor antagonist (mianserin). This may have been caused by functional differences of the octopamine receptor antagonist, and the receptor's blocking effect might have been too strong, such that many injected aphids did not survive (data not shown) and reproduce well after treatment.

**Figure 6:**
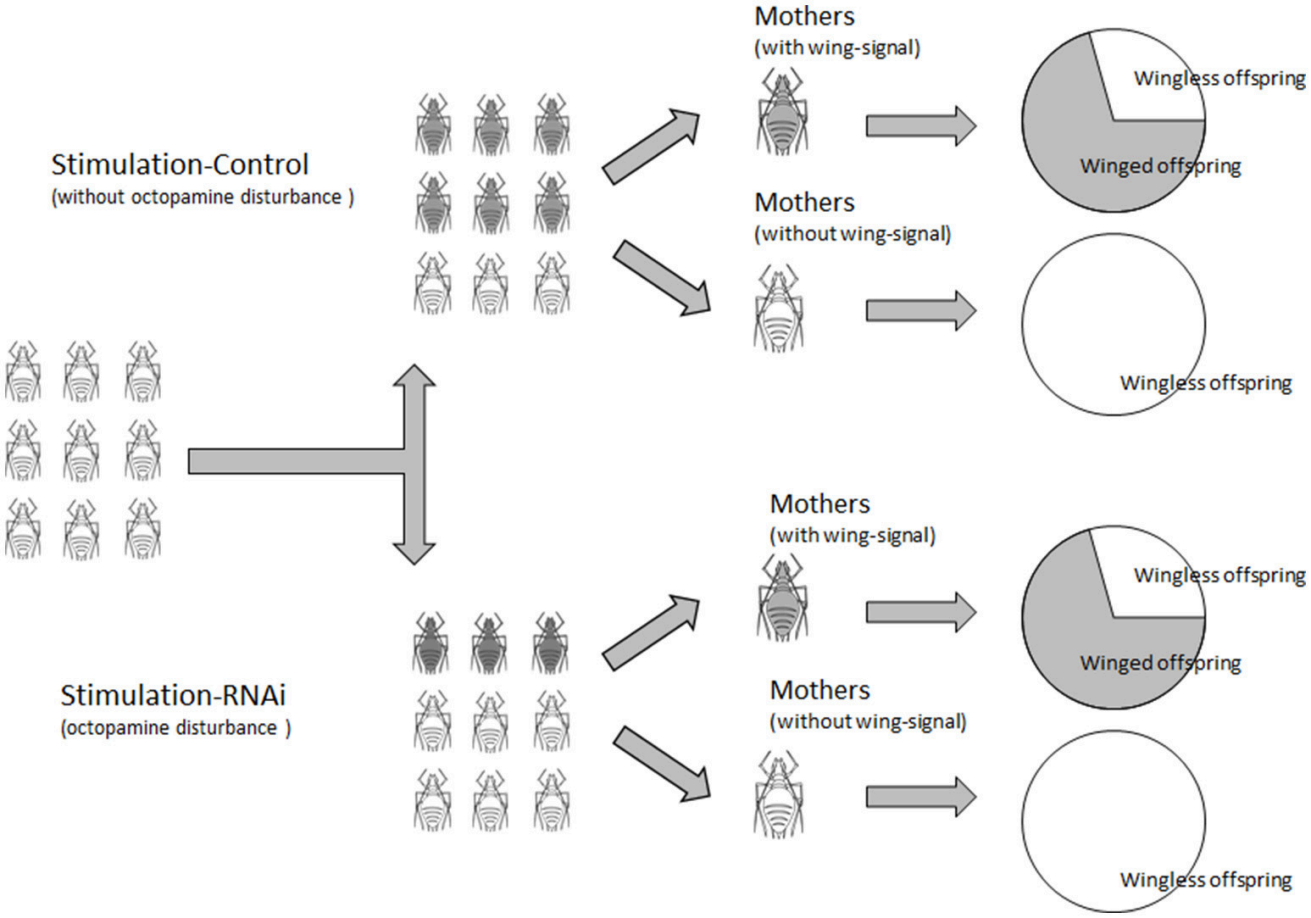
**The hypothesis of octopamine functions in wing formation of ***Acyrthosiphon pisum*****.

Winged nymph stimulation experiments showed that the proportion of winged offspring declined with *T*β*H* RNAi under high-density conditions, which confirmed our hypothesis that octopaminergic signaling was involved in wing polyphenism. We assumed that aphid locomotion would decline when octopamine level declined. Our results showed that body contact among stimulated aphids decreased and fewer winged producers (with wing-formation signal) were stimulated in the *T*β*H* dsRNA treatment. This was supported by the results from the octopamine receptor antagonist injections. It is possible that fewer body contacts among aphids provided the illusion that the density was not high enough to form winged offspring. The highest proportion of winged nymphs was observed in the un-injected aphids; it seems that mechanical or physical injury may influence the mother aphids in some ways, resulting in production of fewer winged aphids. Furthermore, no treated aphids without high density stimulation produced winged daughters, implying that density is important in wing-stimulation. The proportion of winged offspring of successfully stimulated aphids (winged producers) in all treatments was similar, except for the un-injected aphids, which produced more winged offspring. These results indicate that the decline in the proportion of winged offspring was primarily caused by the decline of the proportion of successfully stimulated mothers among injected aphids (Figure 6). Similar to the results of the nymph production experiment, octopamine injection resulted in only slightly higher winged-offspring production than that of the control. These results support the supposition that only a small amount of octopamine passed through the nervous system of the aphids, or bio-degraded quickly. These unexpected results from the non-treated control require further verification.

Wing polyphenism in pea aphids is complicated and can be modified by environmental, biological, genetic situations, and other biotic and abiotic factors. A previous study on the migratory locust showed that octopamine was involved in the regulation of the locust's phase changes (Verlinden et al., 2010; Ma et al., 2015). Octopaminergic signaling in the pea aphid is now confirmed to play an important role in wing formation. The fluctuation in octopamine levels is related to aphid densities, which, in turn, causes different proportions of winged offspring. On the other hand, by modifying octopaminergic signaling, either by down-regulating *T*β*H* expression or by blocking octopamine receptors, the proportion of winged offspring could also be changed. We assume that octopaminergic signaling could be a link in this wing-determination control system, and functions in the mother's generation. Some other physiological characteristics, such as offspring production, could also be affected. Our data also showed that winged nymph production of each mother was not affected by octopaminergic signaling, but fewer winged nymph producers were stimulated. Fewer winged nymph producers caused a decrease in winged offspring in subsequent generations. However, the wing-signal transport pathway from one generation to the next is still unclear in *A. pisum*, and further study is needed.

## Author contributions

XW, HT, YZ, and TL designed research; YZ and XW performed research; HT and ZZ provided assistance; ZZ performed LC/MS analysis; YZ and HT analyzed data; XW, YZ, HT, and TL wrote the paper.

### Conflict of interest statement

The authors declare that the research was conducted in the absence of any commercial or financial relationships that could be construed as a potential conflict of interest.
